# Social-ecological factors associated with selling sex among men who have sex with men in Jamaica: results from a cross-sectional tablet-based survey

**DOI:** 10.1080/16549716.2018.1424614

**Published:** 2018-01-18

**Authors:** Carmen H. Logie, Ashley Lacombe-Duncan, Kathleen S. Kenny, Kandasi Levermore, Nicolette Jones, Stefan D. Baral, Ying Wang, Annecka Marshall, Peter A. Newman

**Affiliations:** ^a^ Factor-Inwentash Faculty of Social Work, University of Toronto, Toronto, ON, Canada; ^b^ Women’s College Research Institute, Women’s College Hospital, Toronto, ON, Canada; ^c^ Gillings School of Global Public Health, University of North Carolina at Chapel Hill, Chapel Hill, NC, USA; ^d^ Jamaica AIDS Support for Life, Kingston, Jamaica; ^e^ Johns Hopkins Bloomberg School of Public Health, Johns Hopkins University, Baltimore, MD, USA; ^f^ Institute for Gender and Development Studies, Mona Campus, University of the West Indies, Kingston, Jamaica

**Keywords:** MSM, stigma, Jamaica, HIV risk, HIV prevention

## Abstract

**Background**: Globally, men who have sex with men (MSM) experience social marginalization and criminalization that increase HIV vulnerability by constraining access to HIV prevention and care. People who sell sex also experience criminalization, rights violations, and violence, which elevate HIV exposure. MSM who sell sex may experience intersectional stigma and intensified social marginalization, yet have largely been overlooked in epidemiological and social HIV research. In Jamaica, where same sex practices and sex work are criminalized, scant research has investigated sex selling among MSM, including associations with HIV vulnerability.

**Objective**: We aimed to examine social ecological factors associated with selling sex among MSM in Jamaica, including exchanging sex for money, shelter, food, transportation, or drugs/alcohol (past 12 months).

**Methods**: We conducted a cross-sectional survey with a peer-driven sample of MSM in Kingston, Ocho Rios, and Montego Bay. Multivariable logistic regression analyses were conducted to estimate intrapersonal/individual, interpersonal/social, and structural factors associated with selling sex.

**Results**: Among 556 MSM, one-third (n = 182; 32.7%) reported selling sex. In the final multivariable model, correlates of selling sex included: individual/intrapersonal (lower safer sex self-efficacy [AOR: 0.85, 95% CI: 0.77, 0.94]), interpersonal/social (concurrent partnerships [AOR: 5.52, 95% CI: 1.56, 19.53], a higher need for social support [AOR: 1.08, 95% CI: 1.03, 1.12], lifetime forced sex [AOR: 2.74, 95% 1.65, 4.55]) and structural-level factors (sexual stigma [AOR: 1.09, 95% CI: 1.04, 1.15], food insecurity [AOR: 2.38, 95% CI: 1.41, 4.02], housing insecurity [AOR: 1.94, 95% CI: 1.16, 3.26], no regular healthcare provider [AOR: 2.72, 95% CI: 1.60, 4.64]).

**Conclusions**: This study highlights social ecological correlates of selling sex among MSM in Jamaica, in particular elevated stigma and economic insecurity. Findings suggest that MSM in Jamaica who sell sex experience intensified social and structural HIV vulnerabilities that should be addressed in multi-level interventions to promote health and human rights.

## Background

Men who have sex with men (MSM) who sell sex include diverse populations who often experience criminalization and intersecting forms of stigma, violence and social marginalization that elevate HIV risks [1–7]. Yet MSM who sell sex have largely been overlooked in epidemiological and social HIV research. MSM are a key population in the global HIV pandemic [8,9], including in Caribbean and Latin American countries [10–12]. In Jamaica, MSM remain at elevated risk for HIV acquisition with a reported HIV prevalence between 28% and 32% [2,12] in comparison with a general population prevalence of 1.7% (95% CI: 1.4–2.0) among reproductive aged adults [13,14]. This prevalence is among the highest in the Caribbean [2,15], and is shaped, in part, by the criminalization of same-sex sexual behaviors. Criminalization of ‘homosexuality’ in Jamaica contributes to stigma and discrimination, family rejection, and a lack of human rights protections across employment, education, and healthcare systems, as well as socially sanctioned violence from community and the police [16–18].

People who sell sex, that is, persons who exchange sex for money or other goods, in general, are a key population that experiences criminalization, rights violations, and violence that elevate their HIV exposure [1,19]. The prevalence of selling sex among MSM varies across contexts, in part due to methodological challenges in sampling a marginalized, and often criminalized, population [1,2,4,12,20–23]. Among MSM (n = 24,051) in 17 Latin American countries, 7.2% reported transactional sex [24]. Other studies report higher prevalence. One-quarter of MSM in Brazil (n = 658) recruited via respondent-driven sampling reported ever receiving payment for sex [4]. In Jamaica, cross-sectional surveys conducted with MSM in 2007 (n = 201) [12] and 2011 (n = 449) [2] identified the prevalence of sex-selling as 35.9% overall [2], and 30.5% and 39.4% among HIV negative and positive persons, respectively [12]. In contexts with pervasive sexual stigma and discrimination, MSM may experience limited access to education and employment, and may lose familial support; this may contribute to poverty, homelessness, and engagement in survival sex work for money in addition to food, rent, shelter, drugs, and/or alcohol [5,6,20,25].

In some low- and middle-income countries, sex-selling among men has been associated with elevated HIV transmission risks [2,24–32]. Yet, scant research has examined the experiences of MSM who sell sex in contexts where both same-sex practices and sex work are criminalized, such as Jamaica [33]. Two prior studies in Jamaica examining sex-selling among MSM have reported different findings regarding sex work and HIV infection risks. A 2007 study found that HIV prevalence did not differ based on sex work involvement [12], whereas a 2011 study reported higher HIV prevalence among MSM involved in sex work (41.1%) compared to other participants (21.0%) [2]. This study [2] used bivariate analyses to identify structural factors associated with ever being paid for sex among MSM, including homelessness and unemployment. Significant knowledge gaps remain, however, regarding factors associated with selling sex, including multivariable analyses that identify independent effects of multi-level factors, and the possible association between sexual stigma and sex-selling among MSM in Jamaica [2]. Stigma is a particularly important area to examine among MSM who sell sex, who may face intersecting stigma, including sexual stigma, and HIV-related stigma, in addition to sex work stigma [4,5,34–38]. Stigma has been identified as a barrier for male sex workers in accessing HIV prevention services [3].

*Perceived sexual stigma,* concerns about rejection and negative treatment by others because of actual or perceived lesbian, gay, or bisexual (LGB) identity, and *enacted sexual stigma*, experiences of acts of violence and unequal treatment based on actual or perceived LGB identity [34,39], profoundly shape the lives of sexually diverse persons. *Internalized homophobia*, feelings of shame and self-blame [40–43], may contribute to mental health challenges. Sexual stigma may limit access to education and employment, threatening economic security [5]. In addition, sexual stigma, sex work, and HIV-related stigma may limit access to sexual health and HIV information, prevention, and testing and care services [44,45]. Both qualitative [46] and quantitative [47–50] studies describe stigmatizing attitudes by university students and health/social service providers towards people living with HIV and lesbian, gay, bisexual, and transgender (LGBT) persons in Jamaica, with the highest levels of stigma directed towards MSM living with HIV [49,50].

In addition to sexual stigma, other ecological factors are associated with selling sex among MSM. Baral and colleagues [3] conducted a review of studies focused on men who sell sex and identified factors associated with sexual risk practices at biological and behavioural, social, and community levels, consistent with a modified social ecological model [51]. The modified social ecological model conceptualizes multi-level domains associated with HIV infection risks, including proximal intrapersonal and interpersonal level risks (e.g. condomless sex) in addition to distal structural level risks (e.g. stigma) [51]. Examining multi-level HIV risks is particularly important among MSM who sell sex. While factors at a structural level (e.g. sexual stigma, economic insecurity) are interconnected and may contribute to increased engagement in sex-selling among MSM, these factors may also be exacerbated among MSM after they begin to sell sex, when they may subsequently experience the intersection of sexual stigma and sex work stigma [3]. Quantitative studies in various global contexts have identified factors associated with sex-selling among MSM, including socio-demographic factors (e.g. age [22], education [22]), individual risk factors (e.g. condomless anal intercourse [4,22,36], HIV knowledge [22], sexually transmitted infections (STI) history [52,53], higher number of sexual partners [1,2], substance use [54]), and social level factors (e.g., forced sex, harassment and violence [1,2,4,36]). Alternately, social cohesion among MSM who sell sex has been identified as a protective factor [3].

There has been less attention to stigma and multi-level ecological factors, particularly at the structural level, among MSM who sell sex in the Caribbean. Our study objective was to test a conceptual model, based on a social ecological framework, examining intrapersonal/individual, interpersonal/social, and structural level factors associated with sex-selling among MSM in Jamaica.

## Methods

### Setting, study design, and participants

We conducted a cross-sectional study with MSM in Jamaica in Kingston, Ocho Rios, and Montego Bay in 2015–2016. Eligibility for participation included: a) self-identification as a gay or bisexual man, or a man who has sex with, or is sexually attracted to, other men; and b) aged 18 years and older.

Kingston is the capital and largest city of Jamaica, with approximately 600,000 inhabitants [55]. Ocho Rios has a smaller population of approximately 10,000 people [56], but has a large influx of tourists. Outside of urban parishes, parishes with significant tourism-based economies have the next highest level of cumulative number of reported HIV cases, including St Ann’s parish, where Ocho Rios is located [57]. We aimed to include geographic diversity and communities most affected by HIV in Jamaica.

We worked with a national community-based AIDS service organization (Jamaica AIDS Support for Life) and a team of seven peer research assistants (PRAs), who identified as lesbian, gay, bisexual, MSM, or other sexually and/or gender diverse identities. PRAs contributed to survey design and recruitment, and administered the survey. We used chain-referral sampling, a form of peer-driven recruitment, often applied to access hidden or hard-to-reach populations [58]. In this process, each participant was given a unique participant identification (ID) number, and at the end of the survey was given up to five coupons with study information and a coupon ID number and invited to refer peers to the study who met eligibility requirements.

The survey was administered in person by PRA using tablets and an online survey with FluidSurveys™ software. Participants provided written informed consent at the time of their interview, and received $1000 Jamaican dollars (approximately $8 USD) for completing the 45-minute survey, and an additional $500 Jamaican dollars (approximately $4 USD) for each participant they successfully recruited to participate. PRA read the informed consent information aloud to the participant from the tablet; before being permitted to complete the survey, eligible participants provided voluntary written informed consent on the tablet. The Research Ethics Board at the University of Toronto and the University of West Indies, Mona Campus, provided research ethics approval for this study.

### Survey measures

#### Sex-selling

Sex-selling was assessed by asking participants if they had exchanged sex for money, shelter, food, transportation, or drugs/alcohol in the last 12 months. Participants who self-reported selling sex for any of the above reasons were coded as ‘yes’ and those self-reporting no sex selling were coded as ‘no’.

#### Socio-demographic factors

We assessed socio-demographic factors including: age (continuous); education level (less than high school/completed high school); city of residence (categories: Kingston, Montego Bay, Ocho Rios, and other); and monthly income (continuous; we report in US dollars).

#### Intrapersonal/individual factors

Intrapersonal/individual factors assessed included: HIV status (positive/negative; assessed by self-report), lifetime STI history (positive/negative, measured by self-report of having received an STI test and the results with a diagnosis), depression symptoms in the past 2 weeks (continuous, measured with Patient Health Questionnaire-2 [59], scale range 0–8, Cronbach’s α = 0.67); resilient coping, measured using the Brief Resilience Scale [60] (continuous six-item scale, range 6–30, Cronbach’s α = 0.66), safer sex self-efficacy, using Kalichman et al.’s (2001) scale for negotiating safer sex (continuous, range: 5–20, Cronbach’s alpha: 0.75), and internalized homophobia, using the 12-item version of the Internalized Homophobia Scale developed by Currie et al. [61] (continuous 12-item scale, range 12–77, Cronbach’s α = 0.50). We also assessed lifetime number of sexual partners (continuous) and consistent condom use in the last 4 weeks (no/yes; participants were coded as ‘yes’ if there was parity in the number of times they reported having sex and the number of times they reported using condoms).

#### Interpersonal/social factors

Interpersonal/social factors included: relationship status (categories: in a relationship, casual dating, no partner, concurrent partners); social support, measured with a scale developed by Bernal et al. [62], which included two subscales: the need for social support (Cronbach’s a = 0.81, range 7–35) and satisfaction with the quality of social support (Cronbach’s a = 0.86, range 2–10); lifetime history of childhood sexual abuse (no/yes), lifetime history of childhood physical abuse (no/yes), and lifetime history of forced sex (no/yes).

#### Structural factors

We assessed HIV-related stigma using Steward et al.’s 10-item perceived stigma subscale (continuous 10-item scale, range 8–100, Cronbach’s α = 0.92). We measured sexual stigma using Diaz et al.’s scale [63] that includes perceived sexual stigma (continuous five-item scale, range 7–35, Cronbach’s α = 0.73) and enacted sexual stigma (continuous seven-item scale, range 7–49, Cronbach’s α = 0.88). We assessed food insecurity (no/yes; participants were coded as ‘food insecure’ if they reported at least one occurrence of going to bed hungry in the past week), unstable housing in last month (no/yes; participants were coded as having unstable housing if they usually slept outside, in a shelter, or at a friend’s or relative’s house vs in their own room in an apartment/house), employment (unemployed vs employed or studying), barriers to healthcare (no/yes; participants were coded as ‘yes’ if they reported one or more barriers to accessing healthcare services); and having a regular healthcare provider (no/yes).

### Statistical analyses

Theoretically important factors mapping onto individual, social and structural levels of the social ecological model [51] were examined as correlates of sex-selling in the last 12 months in bivariable logistic regression analyses using proc genmod with a logit link. Variables with p values of <0.05 were considered for inclusion in the full multivariable logistic regression model. A forward stepwise model-building procedure using logistic regression and Akaike’s Information Criterion (AIC) was used to determine variables for inclusion in the final model. The final model was preferred over reduced models because of the smaller AIC value. Only those variables independently associated with the outcome of selling sex were retained in the final model. All statistical analyses were conducted using SAS software version 9.3 (SAS Institute, Cary, NC, USA).

## Results

### Study population

In Table 1, we present the characteristics of study participants by sex-selling status in the last 12 months. Of 556 MSM participants, 182 (32.7%) reported selling sex in the past 12 months. There were no significant differences in HIV status or lifetime STI history by sex-selling; 12.1% (n = 67) of all participants reported being HIV-positive and 8.8% (n = 49) reported a lifetime STI history.

**Table 1.** Individual, social, and structural factors and sex selling in the past 12 months among men who have sex with men in Jamaica (n = 556).

Characteristic	Sold sex (n = 182)	Did not sell sex (n = 374)	Missing	p-value
**Socio-demographic factors**				
Age, years (*median,* interquartile range (IQR))	25.0 (22.0–27.0)	24.5 (21.0–28.0)	14	0.9513
Education, less than high school	48 (26.4)	30 (8.0)		<0.0001
Monthly income USD (*median, IQR*)	78.4 (0.5–188.1)	156.7 (39.2–313.5)	20	<0.0001
Location (city)				
*Kingston*	37 (20.3)	118 (31.5)		ref.
*Montego Bay*	41 (22.5)	84 (22.5)		0.0986
*Ocho Rios*	99 (54.5)	112 (30.0)		<0.0001
*Other*	5 (2.8)	60 (16.0)		0.0083
**Intrapersonal/individual factors**				
HIV status (positive)	25 (15.7)	42 (12.4)		0.3099
Lifetime STI history	19 (14.1)	30 (11.2)		0.3967
Depression symptoms (*median, IQR*)	6.0 (5.0–7.0)	5 (4.0–6.0)		<0.0001
Coping – resilience (*median, IQR*)	18 (17–21)	19 (17–23)		0.0424
Safer sex self-efficacy	18 (16–20)	19 (17–20)		<0.0001
Internalized homophobia (*median, IQR*)	46.5 (41.0–53.0)	48.0 (43.0–54.0)		0.0344
Lifetime sexual partners (*median, IQR*)	18 (8–50)	10 (5–20)	20	0.0003
Inconsistent condom use	40 (22.00)	50 (13.4)		0.0098
**Interpersonal/social factors**				
Relationship status				
*In a relationship*	77 (42.3)	202 (54.3)		ref.
*Casual dating*	38 (20.9)	66 (17.8)		0.0831
*No partner*	40 (22.0)	98 (26.3)	2	0.7342
*Concurrent partnerships*	27 (14.8)	6 (1.6)		<0.0001
Social support (need) (*median, IQR*)	26 (22–30)	22 (18–26)		<0.0001
Social support (satisfaction) (*median, IQR*)	6 (5–8)	8 (6–9)		0.0008
Childhood sexual abuse	60 (33.0)	52 (13.9)		<0.0001
Childhood physical abuse	74 (41.3)	17 (19.1)	5	<0.0001
Experienced forced sex in lifetime	110 (60.4)	90 (24.1)		<0.0001
**Structural factors**				
HIV stigma (*median, IQR*)	79.5 (68.0–89.0)	69 (56.0–83.0)		0.0004
Perceived sexual stigma (*median, IQR*)	17 (14–18)	14 (11–16)		<0.0001
Enacted sexual stigma (*median, IQR*)	15 (11–19)	9 (7–13)		<0.0001
Food insecurity	129 (70.9)	137 (36.7)	1	<0.0001
Unstable housing	85 (50.0)	90 (24.8)	23	<0.0001
Currently employed or studying	114 (64.8)	284 (77.8)	15	0.0013
Experience 1 or more barriers to healthcare access	129 (70.9)	156 (41.8)		<0.0001
Do not have a regular healthcare provider	135 (74.2)	186 (49.7)		<0.0001

### Logistic regression modeling of sex-selling among MSM in Jamaica

Factors associated with sex-selling among MSM in bivariate and multivariable analyses are displayed in Table 2. In bivariate analyses, socio-demographic factors correlated with sex-selling at alpha <0.05 included: lower likelihood of completing high school education, lower income, lower likelihood of living in an ‘other’ area vs Kingston, and higher likelihood of living in Ocho Rios vs Kingston. Intrapersonal/individual factors correlated with sex-selling at alpha <0.05 included: higher depression symptoms, lower resilient coping, lower safer sex self-efficacy, lower internalized homophobia, and greater odds of inconsistent condom use. Interpersonal/social factors correlated with sex-selling at alpha <0.05 included: higher likelihood of concurrent partnerships vs a relationship, a higher need for social support, lower satisfaction with social support, higher likelihood of childhood physical and sexual abuse, increased odds of lifetime forced sex. Structural factors correlated with sex-selling at alpha <0.05 included: higher HIV stigma, and higher perceived and enacted sexual stigma, food insecurity, unstable housing, current unemployment, one or more barriers to healthcare access, and not having a regular healthcare provider.

**Table 2.** Bivariable and multivariable analyses of individual, social, and structural factors associated with sex-selling in the past 12 months among men who have sex with men in Jamaica (n = 556), March 2015–October 2015.

Characteristic	Unadjusted OR (95% CI)	Adjusted OR (95% CI)
**Socio-demographic factors**		
Education, less than high school	4.11 (2.50, 6.76)**	
Monthly income	0.79 (0.70–0.89)^a^***	
Location (city)		
*Ocho Rios (vs Kingston)*	2.82 (1.78, 4.46)***	2.18 (1.18, 4.04)*
*Other (vs Kingston)*	0.15 (0.09, 0.71)**	0.31 (0.10, 0.95)*
**Intrapersonal/individual factors**		
Depression symptoms	1.40 (1.25, 1.57)^b^**	
Resilience/coping	0.96 (0.92, 1.00)^b^*	
Safer sex self-efficacy	0.82 (0.76, 0.88)^b^**	0.85 (0.77, 0.94)^b^**
Internalized homophobia	0.98 (0.96, 1.00)^b^*	
Inconsistent condom use	1.83 (1.15, 2.89)^b^*	
**Interpersonal/social factors**		
Relationship status		
*Concurrent partnerships* *(vs in a relationship)*	10.92 (4.74, 30.0)***	5.52 (1.56, 19.53)**
Social support (need)	1.13 (1.10, 1.17)^b^***	1.08 (1.03, 1.12)^b^**
Social support (satisfaction)	0.87 (0.80, 0.95)^b^**	
Childhood sexual abuse	3.05 (1.99, 4.66)**	
Childhood physical abuse	2.99 (2.01, 4.43)**	
Experienced forced sex in lifetime	4.82 (3.30, 7.05)**	2.74 (1.65, 4.55)**
**Structural factors**		
HIV stigma	1.01 (1.01, 1.03)^b^*	
Perceived sexual stigma	1.22 (1.15, 1.29)^b^**	
Enacted sexual stigma	1.21 (1.17, 1.26)^b^**	1.09 (1.04, 1.15)^b^**
Food insecurity	4.19 (2.86, 6.15)**	2.38 (1.41, 4.02)*
Unstable housing	3.03 (2.07, 4.45)**	1.94 (1.16, 3.26)*
Currently unemployed	1.91 (1.28, 2.83)^b^**	
Experienced 1 or more barriers to healthcare access	3.39 (2.31, 4.95)**	
Do not have a regular healthcare provider	2.90 (1.97, 4.28)**	2.72 (1.60, 4.64)**

^a^per 100 USD increase.

^b^per 1-unit increase.

*p < 0.05; **p < 0.001; ***p < 0.0001.

In the final multivariable model, socio-demographic correlates of sex-selling included residing in Ocho Rios (AOR: 2.18, 95% CI: 1.18, 4.04) or ‘other’ regions (AOR: 0.31, 95% CI: 0.10, 0.95) in comparison with Kingston. Intrapersonal/individual level correlates of sex-selling included lower levels of safer sex self-efficacy (AOR: 0.85, 95% CI: 0.77, 0.94). Interpersonal/social level factors associated with sex-selling included having concurrent partnerships (AOR: 5.52, 95% CI: 1.56, 19.53), a higher need for social support (AOR: 1.08, 95% CI: 1.03, 1.12), and experiencing forced sex in one’s lifetime (AOR: 2.74, 95% CI: 1.65, 4.55). At the structural level, MSM who sell sex experienced higher enacted sexual stigma (AOR: 1.09, 95% CI: 1.04, 1.15), and twofold higher odds of food insecurity (AOR: 2.38, 95% CI: 1.41, 4.02), unstable housing (AOR: 1.94, 95% CI: 1.16, 3.26), and not having a regular healthcare provider (AOR: 2.72, 95% CI: 1.60, 4.64).

## Discussion

This study applies a social-ecological framework to examine multi-level factors, including sexual stigma, associated with selling sex among MSM in Jamaica. Approximately one-third of participants reported selling sex in the past 12 months. While higher than some other global contexts [4,20,24], this prevalence is similar to prior studies with MSM in Jamaica [2,12]. Our finding that selling sex was not associated with differences in HIV serostatus corroborates findings from a 2011 study with MSM in Jamaica [2], yet contrasts with other global studies [2,26–32]. However, the association of HIV prevalence and sex work among MSM is inconsistent across studies due to a number of factors, including different sex roles of male sex workers in different sociocultural contexts, frequency of condom use, baseline HIV prevalence, and sampling [3]. Nevertheless, MSM in our study overall had sevenfold higher HIV prevalence (12.1%) than the general population in Jamaica [13,14]; and those MSM who sell sex experienced multiple social and structural drivers of HIV that can inform preventive interventions.

**Figure 1.**
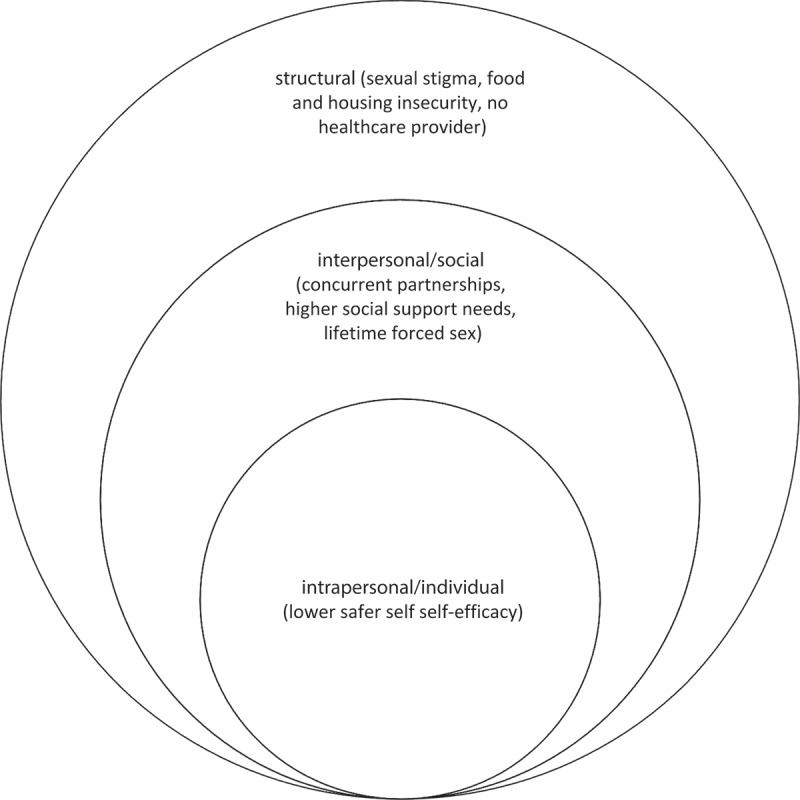
Conceptual model of social ecological factors associated with selling sex among men who have sex with men in Jamaica (n = 556).

We found sex working MSM had greater odds of experiencing a range of HIV vulnerabilities spanning *individual* (e.g. lower safer sex self-efficacy, multiple sex partners), *social* (higher odds of forced sex, need for social support), and *structural* domains (enacted sexual stigma, food and housing insecurity, no regular healthcare provider). These findings can inform the development of tailored interventions to address population-specific HIV vulnerabilities among MSM who sell sex in Jamaica, including a focus on understanding regional differences among MSM who sell sex in Ocho Rios and Kingston. Figure 1 illustrates this conceptual model of social ecological factors associated with selling sex among MSM in Jamaica.

At the individual level, sex-selling was associated with lower odds of safer sex self-efficacy. While only significant in bivariate analysis, it is also notable that MSM who sell sex, in addition to tenfold higher odds of concurrent partners, had nearly double the odds of inconsistent condom use. This contrasts with studies among more formal male sex workers or those affiliated with the gay entertainment industry that indicate higher levels of condom use than among other MSM [13,14]. This difference may reflect the economic insecurity characteristic of the present sample, pervasive forced sex, and the lesser networking with HIV prevention outreach than among other more formal groups of MSM who sell sex [13,14]. In their review of HIV risk factors for young MSM, Mustanski and colleagues [25] suggested several potential mechanisms by which safer sex self-efficacy may be constrained in the context of selling sex, including: lacking essential skills to discuss condom use and HIV/STI with clients; condoms may not be readily available for free and, given other needs (food, shelter, clothing), may not be prioritized for purchase; MSM who are street-based sex workers may not carry condoms due to sex work criminalization and fear of being detained; and the economic insecurity that is often paired with sex work for MSM, which may lead some MSM to accede to clients who may request condomless sex for higher pay. Associations between inconsistent condom use and some of these factors (e.g. economic insecurity, low educational attainment) have been identified in other studies with MSM who sell sex globally [21,36]. Thus, as suggested by Newman and colleagues in studies of MSM who sell sex in other low- and middle-income countries [25], interventions predicated on individual level factors, such as HIV knowledge, condom negotiation and safer sex self-efficacy, would likely be more effective if paired with interventions to increase condom access and address economic security among MSM who sell sex.

At the social level, we identified risk factors for HIV acquisition (forced sex, need for social support) associated with selling sex among MSM. Most (60%) MSM who sold sex in our sample experienced forced sex; these men had almost three times the odds of experiencing forced sex relative to MSM who did not sell sex. Our findings corroborate studies in other contexts that report elevated risks for experiencing sexual and other physical violence among MSM who sell sex [1,2,4,7,36]. This prevalence of forced sex is very high, particularly in comparison to data from other low- and middle-income countries which show prevalence ranging from 6.5 to 40.5% [1,64]. Forced sex among our sample of MSM was also higher than rates of forced sex reported in a prior study with MSM in Jamaica (18.9%) [2]. In a qualitative study with sex workers in Jamaica, which included male sex workers, narratives revealed experiences of violence, including kidnapping, physical abuse, stabbing, robbery, and rape [33]. These findings suggest the need for structural interventions to combat sexual and other physical violence against MSM who sell sex. Future studies may seek to include a measure of sex work social cohesion in addition to social support, which has been linked with increased condom use efficacy among sex workers in Canada [65]. Nevertheless, the potential benefits of social cohesion and social support may be mitigated in the context of criminalization of same-sex behaviors and structural violence, which may exacerbate the risks associated with disclosure of one’s sexual behaviors or identity [65].

Our findings of a significant association between sexual stigma, a structural factor, and selling sex among MSM corroborate Baral et al.’s call for the need to address intersecting stigma among male sex work – associated with HIV, same-sex practices, poorer socioeconomic status, and the criminalization of sex work [3]. Future studies should examine intersectional stigma [66], including sex work stigma, and its association with the health and wellbeing of MSM who sell sex in Jamaica [34].

In addition to sexual stigma, we identified other structural factors among MSM who sell sex that may elevate HIV vulnerabilities. Sex-selling MSM reported higher levels of economic insecurity, with increased unstable housing and food insecurity. Our finding regarding housing insecurity corroborates a prior study with MSM in Jamaica, whereby homelessness was associated with ever being paid for sex in bivariate analyses [2]. Similarly, Oldenburg et al. [24] reported unemployment was associated with increased odds of selling sex in a review of MSM across 17 Latin American countries. Enacted sexual stigma may be a driver of economic insecurity and contribute to sex-selling; enacted sexual stigma scale items include losing a place to live or a job/career opportunity due to one’s sexual identity. Future studies that use longitudinal designs can better ascertain the relationships between enacted sexual stigma, economic insecurity, and sex-selling among MSM in Jamaica.

A particularly concerning finding was that sex-selling MSM were almost three-fold more likely to not have a regular healthcare provider relative to MSM who do not sell sex. MSM who sell sex may actively avoid healthcare, as well as HIV testing, due to anticipated stigma: a survey with 332 staff of healthcare and social service agencies in Jamaica and the Bahamas found that while most respondents said they believed that people living with HIV, MSM, and sex workers deserved quality care, they expressed a high level of blame and negative moral judgments towards MSM [50]. A study with MSM (n = 2035) in Latin America [67] reported that transactional sex was associated with reduced engagement in HIV medical care, underscoring the importance of addressing intersectional stigma targeting MSM, sex workers, and people living with HIV. A recent qualitative study [18] exploring HIV testing experiences with young MSM (n = 20) and community-based key informants (n = 13) in Kingston, Jamaica found that experiences of perceived and enacted stigma in healthcare settings due to sexual stigma and HIV-related stigma presented barriers to accessing HIV testing and sexual healthcare. This suggests the importance of structural level interventions that promote access to healthcare, and reduce discrimination in healthcare against MSM, including those who sell sex, as a crucial measure to intervene in the HIV epidemic among MSM in Jamaica.

There are several study limitations. The cross-sectional study design limits understanding of causality and the nature of relationships between sex-selling and ecological factors. To access this marginalized population we utilized non-random sampling; this limits the ability to generalize findings across MSM in Jamaica. Chain referral sampling could introduce bias in the sample: the initial persons recruiting may not be representative of the original sample and there may be short recruitment chains; additionally, the monetary incentive could appeal to MSM with lower income [68]. This study is specific to MSM, thus we cannot extrapolate findings to other male sex workers who may sell sex to men and women but not identify as gay or bisexual [3]. Behavioral questions, such as condom use, may be subject to social desirability bias whereby participants may have over-reported condom use. HIV status was measured using self-report; we may have underestimated HIV prevalence due to stigma/confidentiality concerns. Future research should use point-of-care testing to more accurately assess HIV seropositivity, and any association with sex-selling. Despite these limitations, this is among the first studies to examine the association between ecological factors spanning multiple domains with selling sex among MSM in Jamaica. Study findings can inform interventions and future research with this population.

## Conclusions

There is a dearth of intervention studies tailored for the needs of MSM who sell sex [3]. Future studies could aim to culturally adapt and rigorously evaluate evidence-based HIV prevention and care interventions for MSM in Jamaica. Interventions for MSM who sell sex could integrate and evaluate successful components of community mobilization interventions with female sex workers as recommended by the Global Network of Sex Work Projects [69] and outlined in several sources, including the Sex Worker Implementation Tool (SWIT) [70]. These include extensive community engagement, political advocacy, building social relationships, and targeted individual support through peer outreach [71]. Given the high rates of forced sex in our findings among MSM who sell sex, interventions can also address community norms and work with state actors (e.g. police) to reduce violence and increase access to justice for MSM in Jamaica [72]. Finally, healthcare providers could benefit from interventions to reduce intersecting stigma to better care for MSM who sell sex [73]. Ultimately, challenging social and structural contexts of stigma and violence targeting MSM – and particularly MSM who sell sex – in Jamaica is necessary to increase health, human rights, and reduce HIV vulnerabilities.

## Paper context

Men who have sex with men (MSM) who sell sex often experience criminalization, stigma, and violence that elevate HIV risk. Limited research has examined the experiences of this population in contexts where both same-sex practices and sex work are criminalized. Our study addresses these knowledge gaps by examining social and structural factors associated with selling sex among MSM in Jamaica, including stigma and economic insecurity. The findings inform multi-level policy and practice intervention approaches.
